# Life history changes in *Trogoderma variabile* and *T. inclusum* due to mating delay with implications for mating disruption as a management tactic

**DOI:** 10.1002/ece3.3865

**Published:** 2018-01-29

**Authors:** Alison R. Gerken, James F. Campbell

**Affiliations:** ^1^ Agricultural Research Service Center for Grain and Animal Health Research USDA Manhattan KS USA

**Keywords:** delayed mating, demography, dermestid beetles, life history, mating disruption, pest control, stored product pests

## Abstract

Controlling postharvest pest species is a costly process with insecticide resistance and species‐specific control requiring multiple tactics. Mating disruption (MD) can be used to both decrease a female's access to males and delay timing of mating and decreases overall mating success in a population and population growth rate. Development of new commercially available MD products requires an understanding of life history parameters associated with mating delay. These can provide information for targeting proportions of reproducing individuals using MD. After delaying mating for females of two closely related beetle species, *Trogoderma variabile* and *T. inclusum*, we surveyed survivorship, number of eggs laid, and number of progeny emerged. With increases in mating age, total number of eggs laid and total number of progeny emerged significantly declined over time. *T. inclusum* typically had greater numbers of eggs laid and progeny emerged compared to *T. variabile* as female age at mating increased, suggesting that *T. inclusum* may be more resistant to long‐term delays in mating. Life span showed an increase as mating age increased but life span significantly decreased almost immediately following mating. Simulations depicting multiple distributions of mating within a population suggest that in a closed population, high levels of mating delay significantly reduced reproductive growth rates. Although reproductive growth rates were decreased with increased mating age, they are still large enough to maintain populations. This study highlights the differences in life history between two closely related species, suggesting that *T. inclusum* outperforms *T. variabile* over the course of a life span, but *T. variabile* has better reproductive capabilities early in life. MD may also be a viable component of a pest management system for these two species as it significantly decreased overall reproductive output and population growth.

## 1 INTRODUCTION

**Figure 1:**
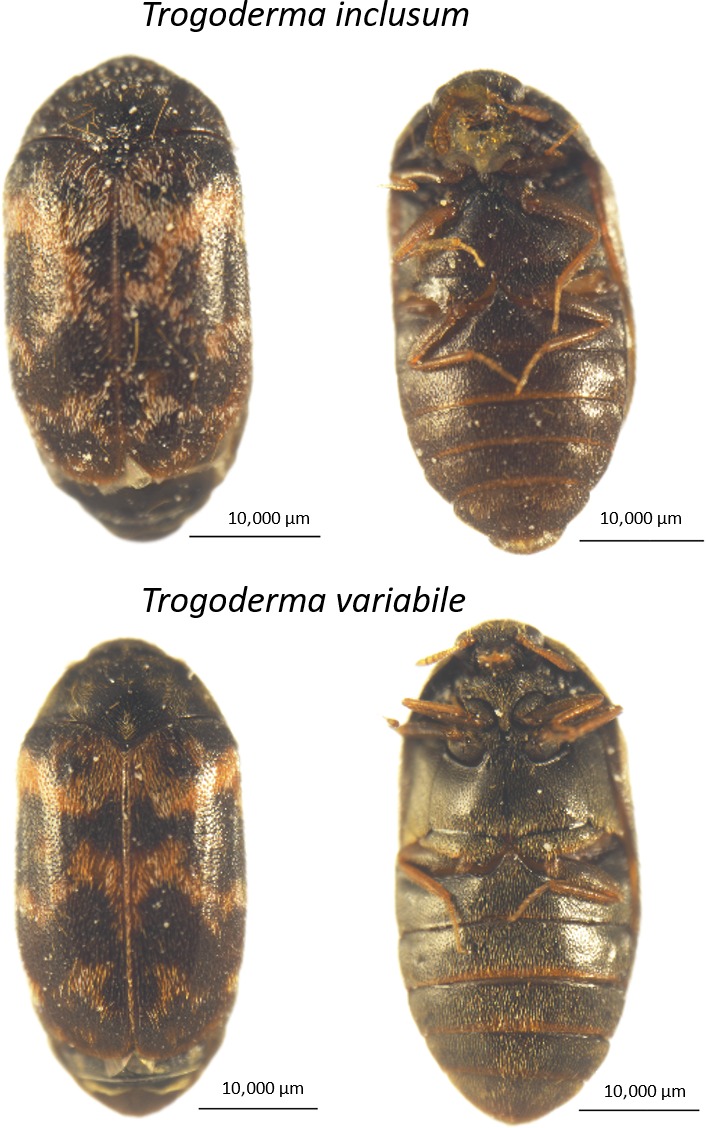
Photographs of *Trogoderma variabile* and *T. inclusum* females

Mating disruption (MD) is a pest management technique where the ability of males and females to access each other is interfered with via competitive, false‐trail following, induced allopatry, and induced arrestment (Miller & Gut, 2015; Miller, Gut, De Lame, & Stelinski, 2006b; Miller, Siegert, Amimo, & Walker, 2009), and/or noncompetitive, desensitization or habituation (Jones & Aihara‐Sasaki, 2001; Kaissling, 1986; Stelinski, Gut, & Miller, 2003a; Stelinski, Miller, & Gut, 2003b), camouflage or when the signal is impaired between males and females (Kuhns, Pelz‐Stelinski, & Stelinski, 2012; Schröder & Hilker, 2008) mechanisms. In recent years, commercial treatments using sex pheromone‐based MD have increased 75%, covering 750,000 hectares across the globe and targeting more than 20 species (Carde & Minks, 1995; Miller & Gut, 2015; Wenninger & Averill, 2006). Commercial MD is also available for management of insects, including *Trogoderma variabile* (Figure 1), that infest stored food inside structures (Barclay & Judd, 1995; Carde & Minks, 1995; Cardé, Staten, & Mafra‐Neto, 1998; Östrand, Wedding, Jirle, & Anderbrant, 1999).

Rather than think of efficacy of MD in terms of whether mating occurs or not, it is more accurate to think of how treatments impact the distribution of times that individuals mate. Complete disruption occurs if mating is delayed to where all individuals die or are no longer reproductively viable. However, under a MD treatment, some individuals may still be able to find each other and mate due to temporal or spatial gaps in treatment (e.g., pheromone concentration) or random encounters. These matings are likely to occur later than under optimal conditions, resulting in a shift in mating times and a corresponding reduction in fitness (Huang & Subramanyam, 2003). Delaying mating has been shown to decrease number of eggs laid, percent of eggs that are fertile, and increase time between mating and egg laying (Jones & Aihara‐Sasaki, 2001; Mori & Evenden, 2013; Sgro & Partridge, 1999). In Indianmeal moths, for example, number of spermatophores per female decreased as mating age increased and egg viability decreased by 22% per day according to female mating age (Huang & Subramanyam, 2003). The combination of effects due to not mating at all and increased age of mating can both contribute to decreasing populations (Cardé, 2007; Jones & Aihara‐Sasaki, 2001; Sanders, 1997; Wyatt, 1997). Models of life history outcomes can include different ranges of effectiveness in MD as well as effectiveness of mating delay on multiple generations (Kiritani & Kanoh, 1984).

Populations contain a mixture of mated and unmated females, which can result from physiological receptivity, lack of opportunity, or infertility defects. For example, 26%–28% of *Neodiprion setifer* females were mated at any given time (Östrand et al., 1999), although specific mechanisms are not known. Flexibility in timing of mating is important for population survival, as availability of mates or competition may delay mating naturally (Carde, 1990; Jones & Aihara‐Sasaki, 2001). Many insects have demonstrable optimal mating times (Ellis & Steele, 1982; Kawazu, Shintani, & Tatsuki, 2014; Proshold, Karpenko, & Graham, 1982; Spurgeon, Raulston, Lingren, & Shaver, 1997). For example, *Cnaphalocrocis medinalis* sex pheromone production increased from ages 2 to 4 days (Kawazu & Tatsuki, 2002) with delayed mating of 7 or 9 days significantly decreasing reproductive output (Kawazu et al., 2014). In comparison, *Lymantria dispar* mate immediately upon pupal emergence and by 3 days of age, the percent of successful mating significantly decreases (Proshold, 1996). Given that in a population, there is a distribution of mating times for females, understanding how fitness is impacted by time of mating and how a treatment such as synthetic pheromone associated with a MD program can shift this distribution of mating times can provide insight into how much of a shift is needed before population growth is negatively impacted.

**Figure 2:**
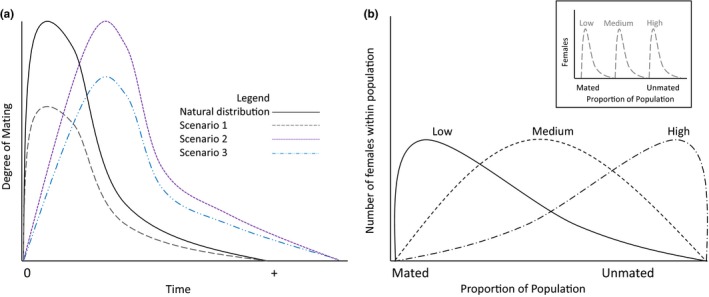
Projected distributions as affected by mating disruption (MD). (a) Theoretical distributions of degree of mated females over time. The natural distribution of mating over time is represented with a high peak in mating early after eclosion as adults with a tail over time. Scenario 1: MD leads to an overall decrease in number of individuals mating at any given time, that is, noncompetitive disruption. Scenario 2: MD causes a shift in optimal mating time, that is, competitive MD. Scenario 3: a combination of competitive and noncompetitive disruption. (b) Theoretical distribution of females within a population that are mated or unmated. There are three types of distributions of control in terms of mated to unmated individuals (Low, Medium, High control), which could be due to MD, at two different levels of skew (inset, gray; main, black). Inset: distributions are narrower and distinct from one another. As we move to the right along our *x*‐axis, we exclude insects that experience no mating delay (mated). Main graph: broad distributions that include mated and unmated females at any given level of MD

Theoretically, effects of MD on a population can shift the distribution of mated females in different ways. Scenario 1 (Figure 2a) follows a distribution where the degree of mating is decreased over time, but there is no shift in timing of mating. This is a possible outcome of a noncompetitive model of MD (Miller et al., 2006b; Stelinski, Miller, & Rogers, 2008) where females are camouflaged by disruption sensory signals that are jamming males’ attraction to females, temporarily “removing” males long enough that they die before they mate, decreasing overall numbers of females mated across time. In Scenario 2, optimal mating time is delayed, reducing the number of progeny, but the number of individuals mating is not reduced. This could occur if it is more difficult for males to find females when a disrupting agent is present due to competitive mechanisms of MD such as false‐trail following (Miller, Gut, De Lame, & Stelinski, 2006a; Miller et al., 2006b). In Scenario 3, some males are removed from the population via jammed signaling (noncompetitive), other males experience false‐trail following (competitive), but there is still mating that occurs by chance or gaps in MD coverage (i.e., wind current changes). This may occur at large densities of insects where competitive and noncompetitive disruption are often indistinguishable from one another (Miller et al., 2006a, 2006b) and result in both a shift in optimal mating timing and a decrease in the number of mating males. In addition, efficiency of control (Figure 2b) can vary based on different factors that impact effectiveness of MD: for example, number of dispensers, gaps in coverage, nonchemical, or visual location of mates (Levinson & Hoppe, 1983). The inset graph (Figure 2b) reflects a stable and consistent treatment, where MD provides a predictable and narrow level of control, which could effectively lead to enough control to negatively impact reproductive characteristics (e.g., net reproductive rate) and future persistence of populations. However, and possibly more realistically, efficiency of control is not as narrow and stable (Figure 2b) creating distributions where there are enough females still producing progeny that population persistence is not controlled by MD techniques (Jones & Aihara‐Sasaki, 2001; Proshold et al., 1982; Spurgeon et al., 1997).

Many dermestid beetle species are important pests of stored food and other materials worldwide and includes the khapra beetle, *Trogoderma granarium* Everts, which is very destructive and is a quarantine pest in the US (Lindgren & Vincent, 1959; Lindgren, Vincent, & Krohne, 1955; Rees, Starick, & Wright, 2003). *Trogoderma variabile* Ballion (Figure 1) is one of the most prevalent and damaging of dermestid species found in the United States (Campbell & Mullen, 2004; Campbell, Mullen, & Dowdy, 2002; Loschiavo, 1960) and is a potential target for the development of MD using sex pheromone as a control tactic. *Trogoderma inclusum* LeConte (Figure 1) is a less damaging pest species that can co‐occur with *T. variabile*. The two species have similar life history and demographic patterns, laying similar numbers of eggs with reported life expectancies that mirror one another (Partida & Strong, 1975; Strong, 1975) and are found across North America (Partida & Strong, 1975; Strong & Okumura, 1966), Australia, Europe, and temperate regions of Asia (Rees et al., 2003). A pheromone for *T. inclusum* is not currently commercially available, but the chemical compound used in *T. variabile* pheromone lures is also a pheromone component for *T. inclusum* and some response by *T. inclusum* to this pheromone component alone has been reported (Greenblatt et al., 1977). In addition, there is a lack of information on mating delay in dermestid beetles (Mori & Evenden, 2013), and this study will provide a foundational basis for application of mating disruption techniques on overall life history and reproductive capacity.

Here, we assessed overall life history characteristics of *T. variabile* and *T. inclusum* due to increased female mating age. We predicted that with an increased mating age, both species will have an increase in survivorship but will have decreases in numbers of eggs laid and progeny emerged and that these closely related species will have similar responses to mating age. If they are different, this would suggest that we need to consider specific life history characteristics when examining pest management goals. We also used Leslie Projection Matrices (Leslie, 1945; Stark, Banks, & Acheampong, 2004) and survivorship‐ and progeny‐based population simulations to make projections to future generations of insects. Finally, we used our laboratory data to model different proportions of mated and unmated females in a population to assess more realistic distributions created by MD within a population and the effects on life history characteristics.

## 2 METHODS

Pupae of *Trogoderma variabile* and *T. inclusum* were collected from stock populations held at 30°C and 60% relative humidity with a 16‐light/8‐hr dark cycle. Stock populations were maintained on a diet of ground dog food and oatmeal with the stock population of *T. variabile* under culture in the laboratory at least 20 years and *T. inclusum* collected from north‐central Kansas in August 2012. Pupae were held in individual 3.7‐ml shell vials with each vial containing 20 mg of organic unbleached, unenriched, all‐purpose flour with 5% brewer's yeast added and sealed with a cotton stopper. Vials were checked each day for adult emergence and once adults emerged, they were held in the same vial until they reached the desired experimental age: 1, 2, 3, 4, 5, 10, or 15 days of age for *T. variabile* and 1, 2, 3, 4, 5, 15, and 29 days of age for *T. inclusum* (differences between species due to results of preliminary tests). All males were 1 day of age at time of pairing. Twenty single‐pair male and female replicates per age group were established. Blocks of females of at least five and up to 14 females were established as females emerged from pupae. These blocks of females were held until their mating status was initiated. For an assessment of survivorship without mating (controls), individual females and males (2–3 in each block) were also established.

Pairs of males and aged females, and single female or male controls, were placed in 30‐ml shell vials containing 1.75 g of presifted (70 mesh) flour and a 1 cm × 5 cm strip of filter paper and then sealed with a cotton stopper. Insects were transferred every 2 days to a new vial with fresh flour until the female died. Two days after adults were transferred out of the vial, the flour was sifted on a 60‐mesh sieve, and eggs were collected and counted. Collected eggs were placed in a 236.6‐ml (1/2 pint) mason jar with 20 g of flour and a filter paper lid. Jars were incubated under the same conditions as stock populations, and adults were counted upon emergence.

For each female mating age group, we summarized data collected for: total number of eggs laid, number of days eggs were laid (oviposition period), and total adult progeny. We calculated life history statistics including survivorship (*l*_*x*_) for each age class within each female age group, mortality (*d*_*x*_), daily mortality rate (*q*_*x*_ = *d*_*x*_/*l*_*x*_), mean reproductive rate per day for each age class (individual fecundity, *m*_*x*_), and net reproductive rate (*R*_o_ = Σ *l*_*x*_ *m*_*x*_). To test for statistical differences in *R*_o_ between female ages both within and between species, we performed a jackknife procedure, where we dropped each of the 20 replicates individually and recalculated *R*_o_ to get a 95% confidence interval. Life history tables were used in Leslie Projection Matrices to infer population growth to 15 generations and population growth rate (lambda (λ)) using the *popbio* package in R (R Statistical Software). We used *m*_*x*_ and *l*_*x*_ for values for fertility (*F*_*i*_) and values for survival from one age to the next (*P*_*i*_) in the matrices, respectively.

To examine differences in mating age between species, we used a generalized linear mixed model using PROC GLIMMIX in SAS (SAS Institute, Cary NC, version 9.4) with our three response variables with main effects of female age, species, and interaction terms for female age and species. Biological replicate and block were used as random effects. Control beetles were not used in this model. Least squares means were calculated for the interaction effect with pairwise comparisons between species made for female age.

We analyzed each species individually to assess differences in mating age within each species. As most of our traits are correlated with one another, we used an individual PROC GLM (SAS version 9.4) for each response variable independently. Main effects in our model were age of female, block, and replicate, with block and replicate being random effects. Means for each age of the female were compared using a Tukey HSD in the model. This model was run without control beetles.

To assess the impact of mating on survivorship, we compared control female survivorship to mated female survivorship at each female age. Each block of mating ages had 2–3 control females assigned to the group; these females did not differ in survivorship across blocks to which they were assigned (*p* = .27, for *T. inclusum*;* p* = .62, for *T. variabile*) so we pooled all control females for comparison with different mating ages. We used PROC GLM with mating age or control status as the main effect. Contrasts were calculated between females of each mating age and control females.

We modeled impacts of mating age using laboratory collected data and created distributions of age at first mating for females within a population to assess effects of different levels of mating age on population outcomes. We do not explicitly know what the distributions of mating times for females within populations of *T. variabile* and *T. inclusum*, but we can choose representative ranges of proportions of mating females within a population. We chose five levels of mating response within a population of 1,000 imputed females. For *T. variabile,* the five levels of population mating delay were as follows: (1) Limited Delay: 80% of the population had survivorship and progeny counts as if they did not undergo mating delay (Day 1 mating), 5% with survivorship and progeny counts as if mating age was 5 days, 5% where mating age was 10 days, 5% where mating age was 15 days (Day 15 mating), and 5% where there was no mating (controls). (2) Low Delay: 50% at 5 days old, 30% at 10 days old, 10% at 15 days old, and 10% no mating. (3) Medium Delay: 10% at 5 days old, 30% at 10 days old, 50% at 15 days old, and 10% no mating. (4) High Delay: 10% at 5 days old, 10% at 10 days old, 30% at 15 days old, and 50% no mating. (5) 80% no mating: 5% at 5 days old, 5% at 10 days old, 10% at 15 days old, and 80% no mating. For *T. inclusum,* we used 29 days of age instead of 15 days. We used our laboratory data on survival proportions for each time step as the basis for imputing population of 1,000 females (Table S1). To impute progeny counts for the population, we used PROC MI in SAS (starting seed = 42,037,621, nimpute = 1, mcmc function) to simulate the appropriate percentage of progeny based on mating age group. We performed a jackknife significance procedure to assess confidence intervals, dropping a replicate from each of the mating ages within the population until we reached the mating age group with the lowest number.

## 3 RESULTS

**Figure 3:**
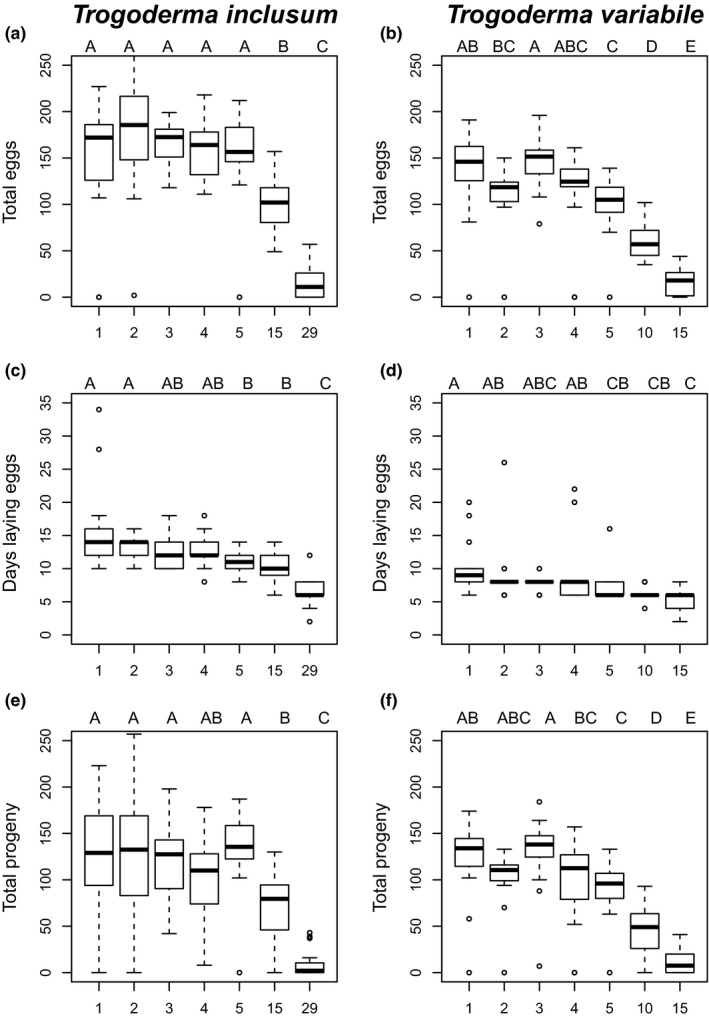
Box plots of measured traits for *Trogoderma inclusum* (left) and *Trogoderma variabile* (right). Tukey pairwise comparisons for analyses within each species are located above each graph. Total eggs laid over the life span of *T. inclusum* (a) and *T. variabile* (b). Number of days that females laid eggs for *T. inclusum* (c) and *T. variabile* (d). Total number of progeny emerged over the life span of *T. inclusum* (e) and *T. variabile* (f)

Total number of eggs laid in a female's life span provides an estimate of their total fecundity potential. *Trogoderma variabile* and *T. inclusum* did not differ in total number of eggs laid when females’ mating age was 1 or 3 days (*p* = .18 and *p* = .094), but *T. inclusum* laid significantly more eggs than *T. variabile* when mating age was 2, 4, 5, or 15 days (*p* ≤ .004; Table S2; Figure 3a–b). At a mating age of 29 days, *T. inclusum* laid a similar number of eggs as *T. variabile* at 15 days mating age (Table S3). Within each species, mating age was a significant factor (*p* < .0001; Table 1), but significant reductions only occurred after mating ages of 15 or 29 days for *T. inclusum* (Table S4; Figure 3a) and after mating ages of 10 or 15 days for *T. variabile* (Table S5; Figure 3b).

**Table 1:** ANOVA table for comparison of *T. variabile* and *T. inclusum*

Effects	Total eggs		Number days laying eggs		Total progeny		Average progeny over life span	
	*F*	*p*‐value	*F*	*p*‐value	*F*	*p*‐value	*F*	*p*‐value
Between species								
Female age	35.33	<.0001	10.91	<.0001	24.73	<.0001	17.31	<.0001
Species	98.65	<.0001	117.50	<.0001	14.57	.0002	0.06	.8066
Female age*species	5.4	.0001	0.64	.6713	4.68	.0004	9.74	<.0001
Within *T. inclusum*								
Female age	36.15	<.0001	13.39	<.0001	18.48	<.0001	11.36	<.0001
Block	0.16	.85	3.78	.025	1.10	.34	0.80	.45
Replicate	0.69	.84	0.67	.85	0.75	.77	0.45	.98
Within *T. variabile*								
Female age	45.14	<.0001	6.34	<.0001	37.97	<.0001	27.54	<.0001
Block	1.38	.26	0.72	.49	1.18	.31	0.10	.90
Replicate	0.99	.48	1.21	.25	0.99	.48	1.12	.34

Denominator DF = 212; numerator DF = 5 for female age and the interaction effect and 1 for species. Within each species, DF = 6 for female age, 2 for block, and 21 for replicate.

Total number of days females laid eggs allows an estimate of mating age impacts on oviposition period duration independent of total number of eggs laid. Age at mating and species were both significant (*p* < .0001) in our overall model, but the interaction term of female age and species was not significant (*p* = .67; Table 1; Figure 3b–c). At each mating age, *T. inclusum* had a longer oviposition period than *T. variabile* (*p* ≤ .0002; Table S2). *Trogoderma inclusum* at mating age of 29 days laid eggs over a similar period of time as *T. variabile* after mating age of 10 days, suggesting that *T. variabile* is more susceptible to long‐term MD than *T. inclusum*. Within each species, number of days a female laid eggs was significantly different for both *T. variabile* and *T. inclusum* (*p* < .0001); the random effect of block, where block is females that started their mating on the same day, was significant for *T. inclusum* (*p* = .025; Table 1). For *T. inclusum* (Table S4), decline in days laying eggs was gradual, with differences starting to occur at 5 days and only a mating age of 29 days being significantly fewer than all other mating ages (Figure 3b). *Trogoderma variabile* females showed a similar gradual decline, with mating age of 5 days or more being significantly shorter in duration than a mating age of 1 day (Table S5; Figure 3c).

Total number of progeny provides a measure of whether the viability of eggs is also affected by mating age. The total number of progeny that emerged was significantly different between species and the interaction between mating age and species was significant (*p* = .0004; Table 1). *T. inclusum* and *T. variabile* females did not differ in total number of progeny after mating ages of 1, 3, and 4 days, but for mating ages of 2, 5, and 15 days *T. inclusum* had a greater number of total progeny (Table S2; Figure 3i–j), with total numbers of progeny being more similar between a mating age of 29 days for *T. inclusum* and 15 days for *T. variabile* (Figure 3i–j). Number of total progeny was also significantly different for mating age within each species (Table 1). For *T. inclusum*, number of progeny did not change out to mating age of 5 days, but then decreased at 15 days and was lowest at mating age of 29 days (Table S4; Figure 3i). Similarly, females of *T. variabile* had significantly less total progeny after a mating age of 10 or 15 days compared to mating age of 1–5 days (Table S5; Figure 3j).

To predict the overall effect on population growth due differences in mating age, we calculated life history tables using collected progeny counts and life span data. Survivorship, mortality, daily mortality rate, and mean reproductive output were calculated for every age class for each mating age mating delay time of females (Table S1). Age class is defined as each day where females were placed in new vials and subsequently progeny was counted. Regardless of mating age, females had the highest mean reproductive output (*m*_*x*_) on the first and second days that they mated. Females also tended to have higher daily mortality rates (*q*_*x*_) between days 4 and 7 postmating.

**Figure 4:**
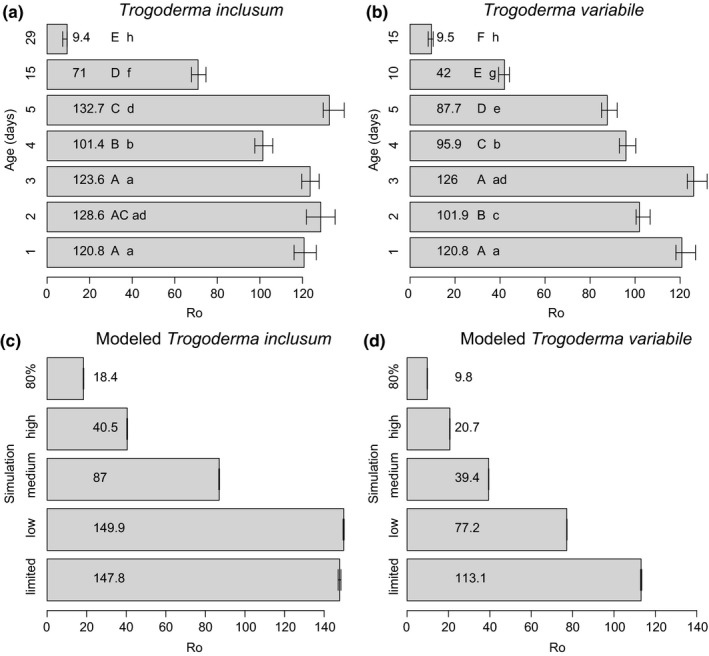
Basic reproductive rate (*R*_o_) for *Trogoderma inclusum* and *T. variabile*, laboratory data (a–b) and modeled (c–d). For (a) and (b), capital letters mark significant differences within *T. inclusum* or within T. variabile among mating ages. Lower case letters represent significant differences between species among mating ages. Error bars are 95% confidence intervals. (a) *R*_o_ for *T. inclusum* for mating ages of 1–5, 15, and 29 days. (b) *R*_o_ for *T. variabile* for mating ages of 1–5, 10, and 15 days. (c–d) Population simulations (*R*_o_) based on survival and progeny counts. “Limited” is where most individuals mate without delay. “Low” is where most individuals are delayed 5 days, but there is still significant mating within the population. “Medium” occurs when most individuals are mating at levels of 10 days (*T. variabile*) or 15 days (*T. inclusum*) of mating age. “High” refers to a population where the majority individuals are mating at levels that correspond to mating ages of 15 days (*T. variabile*) or 29 days (*T. inclusum*). “80%” refers to a population where 80% of the population does not mate; there is some mating but with delays. All simulations are significantly different within and between species

Basic reproductive rate (*R*_o_) with upper and lower 95% confidence intervals was also calculated (Table S4; Figure 4a–b). For both species, there was no change in *R*_o_ in the first 3 mating ages, but after mating age of 4 days, there is a significant drop in *R*_o_. Between species, female mating ages of 1, 3, and 4 days had similar *R*_o_ values. However, at a mating age of 5 days, *T. variabile* has lower *R*_o_ values than *T. inclusum*. Even at a mating age of 29 days, *T. inclusum R*_o_ was similar to *T. variabile* when mated at 15 days (Table S4; Figure 4a–b).

**Figure 5:**
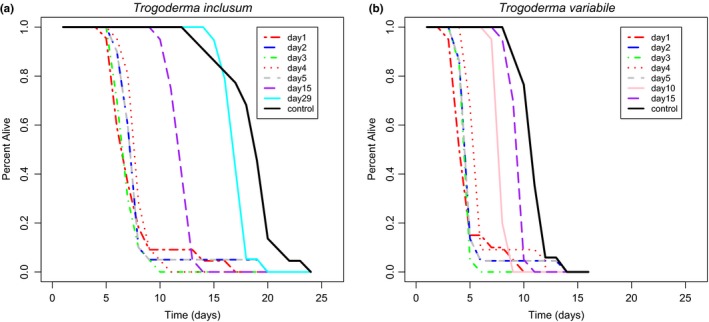
Survivorship curves for *Trogoderma inclusum* (a) and *T. variabile* (b). Survivorship curves were calculated by dividing number of individuals alive at each time point by total number of individuals at the beginning of the experiment. Colors correspond to female age at time of first mating (days), with black indicating survivorship of unmated control females. Mating age significantly increased total life span of experimental beetles for both *T. variabile* and *T. inclusum*; however, mating at any age decreased total survivorship for both species

Assessing survivorship of mated compared to virgin beetle estimates how costly reproduction is for these species. For both species, mated beetles had shorter life spans than control, unmated females (*p* < .0001; Figure 5). Unmated females lived significantly longer than those that were mated at any mating age for *T. variabile* (*p* < .0001 for mating ages 1 to 10; *p* = .0115 for mating age 15). For *T. inclusum*, unmated females lived longer than mated females (*p* < .0001), except after a mating age of 29 days when there was no difference in survivorship from controls (*p* = .0793), where both mated and unmated beetles die soon after this time point. Mated or unmated *T. inclusum* lived longer than mated or unmated *T. variabile* and tended to live longer at all tested mating ages (Figure 5). For both species, mating ages of 1–5 days tended to have similar survivorship curves. Unmated males also had similar life spans as unmated females (data not shown).

**Figure 6:**
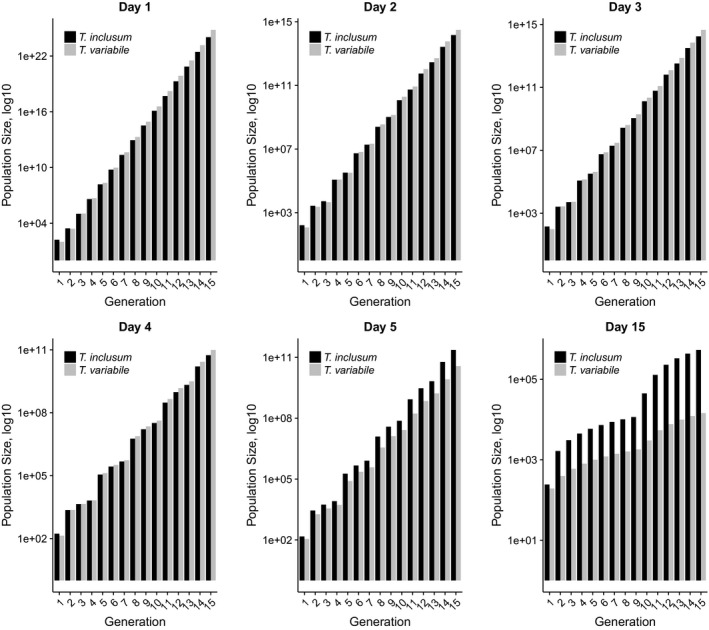
Population size projections. Populations were projected using the *popbio* package in R statistical software to track population sizes 15 generations. Projection factor (i.e., generations) is represented on the *x*‐axis (15 generations). Population sizes (*y*‐axis) have been log_10_ transformed. Mating age for generation 1 only is labeled at the top of each panel. Black bars represent *T. inclusum,* and gray bars represent *T. variabile*

Projecting population sizes following a single generation of mating delay showed that both species’ populations significantly increased in numbers given enough generations. Comparing the two species, *T. variabile* is most successful after 13 generations (of 15 generations projected) when mating age is 4 days or less. However, when mating age was 5 days, *T. inclusum* significantly outnumbers *T. variabile* in projected population size by generation 13 and at mating age of 15 days, *T. inclusum* outnumbers *T. variabile* by generation 4 or 5 (Table S7; Figure 6). This may suggest that *T. inclusum* has a more robust fecundity when faced with a longer‐term mating delay as compared to *T. variabile*. However, when we calculate the finite rate of increase (λ), we find that it is high for both *T. variabile* (44.40) and *T. inclusum* (38.41) when mating age was 1 day. For each increase in mating age, λ decreased but was relatively similar between *T. variabile* and *T. inclusum* which does not explicitly explain projected population size patterns between species (Table S6).

Accounting for variability in mating success within a population and MD success can provide an estimate for overall effectiveness of MD techniques. We found that *R*_o_ values for *T. variabile* were more influenced by changes in mating distribution than *T. inclusum*. There were significant differences between *T. variabile* and *T. inclusum* at all levels of population mating delay, with *T. variabile* having lower *R*_o_ values than *T. inclusum* (Figure 4c–d). Because our population was much larger and values were imputed on 20 individuals for each mating age, confidence intervals were quite small for our simulated *R*_o_ values. For *T. inclusum*, simulated population level mating never got to the lowest level in the collected data (29 days of mating delay; *R*_o_ = 9.4; Figure 4a). With 80% of the population not mating, *R*_o_ was 18.4 suggesting that a population with a distribution of mating and nonmating individuals will have a better overall persistence than a population that is all a mating age of 29 days, which is highly unrealistic. However, for *T. variabile,* a population with 80% of individuals not mating has a similar *R*_o_ outcome to a population where all individuals are of mating age 15 days (*R*_o_ = 9.5; Figure 4b). In addition, *T. variabile* sees a greater decrease in *R*_o_ values between no mating delay and low mating delay than does *T. inclusum*, which actually sees a slight increase in *R*_o_ (Figure 4c–d).

## 4 DISCUSSION

The related insect pest species *Trogoderma variabile* and *Trogoderma inclusum* provide a unique comparison of life history characteristics and an opportunity to model future populations when disrupted via MD programs that have varying levels of impact on mating. This comparative approach offers a look at the narrowness and varying susceptibility of optimal and successful reproduction timing. Delaying optimal timing has significant effects on the biology of these species, and this knowledge can help predict effectiveness of MD. Mating age significantly reduced *R*_o_ for both *T. inclusum* and *T. variabile*, but at no level of mating age examined here was the reduction in progeny and life span enough to successfully decrease *R*_o_ to levels that would eliminate the population.

Similarly, our mating delay simulations yielded populations that would survive to propagate future generations even when 80% of the population did not mate. However, mating delay leads to a decrease in population sizes for several generations even when mating delay occurs in the first generation only. Combined with the fact that MD most likely occurs over several successive generations and natural and warehouse conditions are more stressful, populations where there is a high level of MD may yield sufficient decreases in population sizes from a pest management perspective, even if they do not eliminate the insects. Insect populations with absolutely no mating or extremely low mating likely do not exist due to immigration of already mated females and chance encounters between males and females (Carde, 1990). To achieve a more narrow distribution (Figure 2b, inset) through MD alone may be unrealistic given that the density of pheromone stations would also need to be at unrealistically high levels (Miller et al., 2006b).

In our models, we had a representative percentage of the population that mated at four different levels of mating ages. In pink bollworms, 75% of females were able to mate after less than 1 day delay, only 50% of females were mated at mating age of 5 days (Ellis & Steele, 1982; Lingren, Warner, & Henneberry, 1988; Proshold, 1996) with as few as 23% of females having a spermatophore in Indianmeal moths after a mating age of 5 days (Huang & Subramanyam, 2003). However in other species such as the European grapevine moth, mating success was not affected at mating age of 12 days, with 96%–100% of females mated, but fertilization success significantly reduced (Torres‐Vila, Rodriguez‐Molina, & Stockel, 2002). For *T. variabile* and *T. inclusum,* there is no reduction in successful mating until at least a mating age of 5 days, and *T. inclusum* was less affected by mating age than *T. variabile* with greater age. In addition, when we examine our population size projections for 15 generations (Figure 6), we observed a similar pattern between *T. variabile* and *T. inclusum*; by the time mating age is 5 days, *T. inclusum* significantly outperforms *T. variabile* in population size. By mating age of 15 days, *T. variabile* has no real increase in population size while *T. inclusum* has population growth earlier than for fewer days of delays. This may be due to a longer total life span in *T. inclusum* compared to *T. variabile* and may be a cue or overall benefit in resiliency to mating delay across other species.

At longer‐term delays in mating, *T. inclusum* is also more resistant to overall effects of delays in mating (Figure 4). Life span could be driving this difference, but *T. inclusum* also maintains the ability to lay a larger number of viable eggs than *T. variabile* at 15 days of delayed mating (Table S3; Figure 3). These two factors may go hand‐in‐hand; an extended life span may ultimately lead to the ability of a species to lay viable eggs for a longer period of time as well as adjust their timing of mating to defer negative impacts of a stressful environment which may lead to mating delays. Further physiological analysis is needed to tease apart the differences between these two species, but our data suggest that different mechanisms may be playing a role in combating exposure to mating delays in these two congeneric species.

Our species comparisons suggest that when applying MD as pest control we must consider individual life history characteristics of each species. Understanding when differences in mating age begin to affect reproductive output could help guide determining how amenable a species would be to MD as a pest management program and also help guide what other complementary pest management tactics might be needed to supplement a program. For example, *T. variabile* is a species that has a commercially produced sex pheromone and a relatively short adult life span and appears to be a good target for MD. If we can delay mating greater than 5 days we will most likely begin to reduce numbers of progeny and overall reproductive output. However, if we implement delays that only provide 2–4 days of a delay, we will not have efficient decreases in eggs laid and life span needed to provide a pest management response. Although not a current target for development of a MD program, our results show that *T. inclusum* is predicted to be a less suitable target for MD than *T. variabile*.

As has been shown in other insect species (Campbell, 2005; Proshold et al., 1982; Torres‐Vila et al., 2002), mating significantly reduced life span for both *T. inclusum* and *T. variabile* when compared to unmated females. Oviposition time, total progeny, and total eggs laid were significantly reduced as mating age increased, and optimal mating time was similar for both *T. inclusum* and *T. variabile* (Table S1) (Ellis & Steele, 1982; Kawazu et al., 2014; Proshold et al., 1982; Spurgeon et al., 1997). In addition, predation and environmental factors (e.g., weather) are not accounted for in our model which would enhance natural mortality over time. Even though *T. inclusum* may live longer overall, *T. variabile* is likely to be more competitive under real‐world conditions. *Trogoderma inclusum* may be better able to tolerate lower densities or unfavorable conditions if they survive real‐world conditions long enough. An additional factor not considered in our model is age of the male; increased age of the male also leads to reductions in egg production (Huang & Subramanyam, 2003) and overall hatchability rates (Kawazu et al., 2014), which could lead to increased variability and population control in populations under mating disruption.

The insects used in this study have been under laboratory culture which could influence the responses observed. Field collected insects often show a broader range of timing of mating than laboratory‐reared insects (Huettel, 1976), and other factors occur under field conditions that were not considered in this study, such as previous mating experiences (Adeesan, Rahalkar, & Tamhankar, 1969). Different populations in the field may also have varying levels of heritability and plasticity which may also lead to variation in predicted life history characteristics and may shift our predicted simulation values (Romano et al., 2016). Laboratory‐rearing conditions may also influence mating delay comparisons as laboratory conditions are often at the optimal temperature and humidity to promote best reproductive values with no field‐induced mortality risks present. In this case, our estimates are most likely higher than what would be found in the field, which provides us with a margin of error for targeting mating delay in field populations, although in some cases, laboratory‐reared and field insects have been shown to behave quite similarly (Schwalbe et al., 1974).

Our analyses demonstrate that net reproductive rates of both *T. inclusum* and *T. variabile* are significantly affected by increased mating ages but that decreases in *R*_o_ may not be low enough to control the population through mating delay alone. Using several different methods, we can extend our laboratory data on eggs, progeny, and life span to include modeling population sizes several generations and populations with varying distributions of mated and unmated females. For both species of *Trogoderma*, populations will persist in high numbers even with mating delays implemented with *T. inclusum* showing a stronger resistance to longer mating delays that *T. variabile*. In addition, our models of population‐wide mating delay suggest that we need greater than 80% of females do not mate to reach levels of *R*_o_ that would reflect detrimental effects on future generations. By modeling these population distributions, we can begin to quantify realistic impacts of mating delay due to MD and understand necessary levels of mating delay to manage insect pest populations.

## CONFLICT OF INTEREST

The authors declare no conflict of interest.

## DATA ARCHIVING

Data are archived at the Agricultural Data Commons: https://doi.org/10.15482/USDA.ADC/1416159.

## AUTHOR CONTRIBUTIONS

JFC conceived of design and collected data. ARG analyzed data and wrote manuscript with JFC.
