# Supplementary material for: Life history changes in Trogoderma variabile and T. inclusum due to mating delay with implications for mating disruption as a management tactic
Source: Ecol Evol. 2018 Jan 29;8(5):2428–39. doi: 10.1002/ece3.3865 (PMC5838081; doi:10.1002/ece3.3865)
Supplement: Supplementary file 2 [file ECE3-8-2428-s002.docx]

SUPPLEMENTAL TABLE 1. All life history tables for each mating delay day for *T. variabile* and *T. inclusum*. Column headers are as follows: egg_days=number of days after virgin females were exposed to males. For days with a delay, some egg_days are negative, which accounts for the aging of the female when building the life history tables; number_progeny_Fx=the number of progeny that emerged from the females for eggs laid on the given egg_days; number_alive= the number of females alive at each age class (egg_days); next_age=the number of females alive at the next age class (used for calculating percent_alive at each age class); age=age in days when first mated; age_class=the overall age of the females at each egg_days level; lx=survivorship from one age class to the following age class; dx=mortality or the number dead from a given age class to the next age class; qx=daily mortality rate or dx/lx ; mx=mean reproductive rate per day for each age class or the individual fecundity; lxmx= lx*mx which is used to calculate R_o_ or net reproductive rate; percent_alive=percent of the total females for each species and mating delay age alive at a given age class.

Excel File.

Supplemental Table 2. LSmeans significance between *Trogoderma variabile* and *T. inclusum* at a given female age (day delayed mating). Numerator DF =1 and denominator DF = 212 for all comparisons.

| Variable Analyzed | Female age | F | p-value |
| --- | --- | --- | --- |
| Total eggs laid | Day 1 | 1.75 | 0.1872 |
|  | Day 2 | 33.08 | <0.0001 |
|  | Day 3 | 2.84 | 0.0937 |
|  | Day 4 | 12.93 | 0.0004 |
|  | Day 5 | 22.24 | <0.0001 |
|  | Day 15 | 52.14 | <0.0001 |
| Number days laying eggs | Day 1 | 27.13 | <0.0001 |
|  | Day 2 | 29.09 | <0.0001 |
|  | Day 3 | 18.50 | <0.0001 |
|  | Day 4 | 13.95 | 0.0002 |
|  | Day 5 | 11.06 | 0.0010 |
|  | Day 15 | 21.69 | <0.0001 |
| Average eggs laid over total lifespan | Day 1 | 9.24 | 0.0027 |
|  | Day 2 | 0.28 | 0.6001 |
|  | Day 3 | 15.58 | 0.0001 |
|  | Day 4 | 7.18 | 0.0080 |
|  | Day 5 | 0.21 | 0.6451 |
|  | Day 15 | 29.96 | <0.0001 |
| Total Lifespan | Day 1 | 27.13 | <0.0001 |
|  | Day 2 | 29.09 | <0.0001 |
|  | Day 3 | 18.50 | <0.0001 |
|  | Day 4 | 13.95 | 0.0002 |
|  | Day 5 | 11.06 | 0.0010 |
|  | Day 15 | 21.69 | <0.0001 |
| Total Progeny | Day 1 | 0.07 | 0.7847 |
|  | Day 2 | 3.89 | 0.0500 |
|  | Day 3 | 0.34 | 0.5600 |
|  | Day 4 | 0.02 | 0.8916 |
|  | Day 5 | 10.89 | 0.0011 |
|  | Day 15 | 22.25 | <0.0001 |
| Average progeny over total lifespan | Day 1 | 3.74 | 0.0546 |
|  | Day 2 | 1.72 | 0.1906 |
|  | Day 3 | 5.35 | 0.0217 |
|  | Day 4 | 4.79 | 0.0296 |
|  | Day 5 | 2.02 | 0.1571 |
|  | Day 15 | 31.19 | <0.0001 |
| Percent of eggs emerged to progeny | Day 1 | 3.92 | 0.0491 |
|  | Day 2 | 19.78 | <0.0001 |
|  | Day 3 | 5.67 | 0.0182 |
|  | Day 4 | 12.80 | 0.0004 |
|  | Day 5 | 0.64 | 0.4263 |
|  | Day 15 | 3.97 | 0.0476 |

Supplemental Table 3. Means ± standard deviations for values of *T. variabile* and *T. inclusum* for observed progeny and survivorship traits.

| Species | Mating Delay | Total eggs laid | Egg lay days | Average eggs laid | Total lifespan | Total progeny | Average progeny | Percent eggs to progeny |
| --- | --- | --- | --- | --- | --- | --- | --- | --- |
| *T. variabile* | Day 1 | 136.3 ± 40.65 | 10.0 ± 3.49 | 30.50 ± 10.42 | 10.00 ± 3.49 | 124.50 ± 38.24 | 32.39 ± 12.88 | 91.15 ± 7.28 |
|  | Day 2 | 111.77 ± 28.26 | 8.73 ± 3.98 | 28.65 ± 7.94 | 9.73 ± 3.98 | 104.18 ± 27.25 | 34.39 ± 10.71 | 93.20 ± 6.79 |
|  | Day 3 | 145.7 ± 25.02 | 7.80 ± 0.89 | 37.98 ± 7.87 | 9.80 ± 0.89 | 131.5 ± 36.17 | 42.13 ± 12.36 | 88.50 ± 20.96 |
|  | Day 4 | 118.64 ± 40.99 | 8.55 ± 4.15 | 33.13 ± 12.46 | 11.55 ± 4.15 | 100.14 ± 42.02 | 33.94 ± 15.09 | 83.66 ± 16.89 |
|  | Day 5 | 100.65 ± 29.12 | 7.20 ± 2.28 | 30.28 ± 9.07 | 11.20 ± 2.28 | 91.80 ± 27.69 | 29.59 ± 9.55 | 91.16 ± 6.48 |
|  | Day 10 | 62.30 ± 21.32 | 6.30 ± 0.98 | 20.28 ± 7.78 | 15.30 ± 0.98 | 46.10 ± 27.06 | 15.72 ± 10.06 | 70.71 ± 32.72 |
|  | Day 15 | 17.30 ± 14.25 | 5.40 ± 1.31 | 6.07 ± 4.78 | 19.40 ± 1.31 | 11.15 ± 12.40 | 3.91 ± 4.30 | 57.29 ± 34.70 |
| *T. inclusum* | Day 1 | 151.50 ± 57.77 | 15.36 ± 5.57 | 22.73 ± 9.86 | 15.36 ± 5.57 | 121.09 ± 58.87 | 25.25 ± 12.36 | 79.00 ± 18.51 |
|  | Day 2 | 177.85 ± 56.78 | 14.30 ± 5.78 | 27.29 ± 8.19 | 15.30 ± 5.78 | 128.90 ± 62.77 | 29.54 ± 13.18 | 66.58 ± 24.08 |
|  | Day 3 | 165.50 ± 21.91 | 12.30 ± 2.18 | 27.62 ± 5.64 | 14.30 ± 2.18 | 124.00 ± 42.88 | 33.40 ± 13.34 | 74.08 ± 20.61 |
|  | Day 4 | 159.43 ± 30.67 | 12.38 ± 2.16 | 26.35 ± 4.43 | 15.38 ± 2.16 | 101.81 ± 40.92 | 24.96 ± 12.08 | 62.26 ± 20.72 |
|  | Day 5 | 156.10 ± 43.77 | 10.80 ± 1.64 | 29.07 ± 7.83 | 14.80 ± 1.64 | 134.20 ± 39.00 | 34.96 ± 10.86 | 86.20 ± 8.67 |
|  | Day 15 | 102.20 ± 27.08 | 10.30 ± 1.98 | 20.43 ± 5.85 | 24.30 ± 1.98 | 71.75 ± 33.71 | 25.01 ± 12.90 | 70.33 ± 25.81 |
|  | Day 29 | 16.74 ± 19.07 | 6.53 ± 2.20 | 4.54 ± 4.83 | 34.53 ± 2.20 | 10.05 ± 15.97 | 5.37 ± 9.94 | 40.55 ± 34.85 |

Supplemental Table 4. Tukey’s HSD for number of days laying eggs for *T. inclusum*. Significant difference as calculated by the 95% confidence limit is represented by the * in the “High” column.

| Female age Comparison | Total Eggs Laid | | Number of days laying eggs | | Average Number of Eggs Laid | | Total lifespan | | Total Progeny Emerged | | Average Progeny | | Percent progeny emerged | |
| --- | --- | --- | --- | --- | --- | --- | --- | --- | --- | --- | --- | --- | --- | --- |
| 95% Confidence | Low | High | Low | High | Low | High | Low | High | Low | High | Low | High | Low | High |
| Day 1 – day 2 | -64.66 | 11.96 | -2.23 | 4.35 | -11.45 | 2.32 | -3.23 | 3.35 | -50.17 | 34.55 | -16.11 | 7.53 | -9.26 | 34.09 |
| Day 1 – day 3 | -52.31 | 24.31 | -0.23 | 6.35 | -13.23 | 0.55 | -2.23 | 4.35 | -50.17 | 34.55 | -19.97 | 3.67 | -16.76 | 26.60 |
| Day 1 – day 4 | -45.76 | 29.90 | -0.27 | 6.23 | -10.42 | 3.18 | -3.27 | 3.23 | -22.55 | 61.11 | -12.38 | 10.96 | -4.68 | 38.16 |
| Day 1 – day 5 | -42.91 | 33.71 | 1.28 | 7.85 * | -11.79 | 1.99 | -2.73 | 3.85 | -55.47 | 29.25 | -19.97 | 3.67 | -29.17 | 14.76 |
| Day 1 – day 15 | 10.99 | 87.61 * | 1.78 | 8.35 * | -4.59 | 9.19 | -12.23 | -5.65 * | 6.98 | 91.70 * | -11.58 | 12.06 | -13.01 | 30.35 |
| Day 1 – day 29 | 95.93 | 173.60 * | 5.50 | 12.17 * | 11.20 | 25.16 * | -22.50 | -15.83 * | 68.10 | 153.98 * | 7.90 | 31.86 * | 14.03 | 62.88 * |
| Day 2 – day 4 | -20.32 | 57.16 | -1.41 | 5.25 | -6.02 | 7.91 | -2.37 | 4.37 | -15.75 | 69.93 | -8.38 | 15.53 | -17.10 | 25.75 |
| Day 2 – day 3 | -26.86 | 51.56 | -1.37 | 5.37 | -7.38 | 6.72 | -3.41 | 3.25 | -38.45 | 48.25 | -15.96 | 8.24 | -29.18 | 14.18 |
| Day 2 – day 5 | -17.46 | 60.96 | 0.13 | 6.87 * | -8.82 | 5.28 | -2.87 | 3.87 | -48.65 | 38.05 | -17.52 | 6.68 | -41.58 | 2.34 |
| Day 2 – day 15 | 36.44 | 114.86 * | 0.63 | 7.37 * | -0.18 | 13.92 | -12.37 | -5.63 * | 13.80 | 100.50 * | -7.57 | 16.63 | -25.43 | 17.90 |
| Day 2 – day 29 | 121.36 | 200.84 * | 4.36 | 11.18 * | 15.61 | 29.89 * | -22.64 | -15.82 * | 74.93 | 162.77 * | 11.92 | 36.43 * | 1.61 | 50.46 * |
| Day 3 – day 4 | -32.67 | 44.81 | -3.41 | 3.25 | -5.69 | 8.24 | -4.41 | 2.25 | -20.65 | 65.03 | -4.52 | 19.39 | -9.60 | 33.24 |
| Day 3 – day 5 | -29.81 | 48.61 | -1.87 | 4.87 | -8.49 | 5.61 | -3.87 | 2.87 | -53.55 | 33.15 | -13.66 | 10.53 | -37.83 | 6.10 |
| Day 3 – day 15 | 24.09 | 102.51 * | -1.37 | 5.37 | 0.15 | 14.25 * | -13.37 | -6.63 * | 8.90 | 95.90 * | -3.71 | 20.48 | -17.93 | 25.43 |
| Day 3 – day 29 | 109.04 | 188.49 * | 2.36 | 9.18 * | 15.94 | 30.22 * | -23.64 | -16.82 * | 70.03 | 157.87 * | 15.77 | 40.23 * | 9.11 | 57.96 * |
| Day 4 – day 5 | -35.41 | 42.07 | -1.75 | 4.91 | -9.69 | 4.25 | -2.75 | 3.91 | -75.23 | 10.45 | -20.95 | 2.95 | -45.65 | -2.24 * |
| Day 4 – day 15 | 18.49 | 95.97 * | -1.25 | 5.41 | -1.05 | 12.88 | -12.25 | -5.60 * | -12.78 | 72.90 | -11.00 | 12.90 | -29.49 | 13.345 |
| Day 4 – day 29 | 103.43 | 181.95 * | 2.48 | 9.23 * | 14.74 | 28.86 * | -22.52 | -15.78 * | 48.35 | 135.17 * | 8.48 | 32.71 | -2.48 | 45.91 |
| Day 5 – day 15 | 14.69 | 91.11 * | -2.87 | 3.87 | 1.59 | 15.69 * | -12.87 | -6.13 * | 19.10 | 105.80 * | -2.15 | 22.05 | -6.09 | 37.83 |
| Day 5 – day 29 | 99.64 | 179.09 * | 0.86 | 7.68 * | 17.38 | 31.66 * | -23.14 | -16.32 * | 80.23 | 168.07 * | 17.34 | 41.85 * | 20.98 | 70.34 * |
| Day 15 – day 29 | 45.74 | 125.19 * | 0.36 | 7.18 * | 8.74 | 23.02 * | -13.64 | -6.82 * | 17.78 | 105.62 * | 7.39 | 31.90 * | 5.36 | 54.12 * |

Supplemental Table 5. Tukey’s HSD comparison of 95% confidence limits for *T. variabile*. Significance is indicated by * in the “High” column.

| Female age Comparison | Total Eggs Laid | | Number of days laying eggs | | Average Number of Eggs Laid | | Total lifespan | | Total Progeny Emerged | | Average Progeny | | Percent progeny emerged | |
| --- | --- | --- | --- | --- | --- | --- | --- | --- | --- | --- | --- | --- | --- | --- |
| 95% Confidence | Low | High | Low | High | Low | High | Low | High | Low | High | Low | High | Low | High |
| Day 1 – day 2 | -3.31 | 52.36 | -1.31 | 3.85 | -6.29 | 9.99 | -2.31 | 2.85 | -8.95 | 49.60 | -12.38 | 8.38 | -22.08 | 17.98 |
| Day 1 – day 3 | -37.89 | 19.10 | -0.44 | 4.84 | -15.80 | 0.86 | -2.44 | 2.84 | -36.96 | 22.96 | -20.38 | 0.88 | -17.62 | 22.91 |
| Day 1 – day 4 | -10.17 | 45.50 | -1.13 | 4.04 | -10.77 | 5.51 | -4.13 | 1.04 | -4.91 | 53.64 | -11.94 | 8.83 | -12.79 | 27.75 |
| Day 1 – day 5 | 7.16 | 64.14 * | 0.16 | 5.44 * | -8.10 | 8.56 | -3.84 | 1.44 | 2.74 | 62.66 * | -7.84 | 13.42 | -20.54 | 20.51 |
| Day 1 – day 10 | 45.51 | 102.49 * | 1.06 | 6.34 * | 1.90 | 18.56 * | -7.94 | -2.66 * | 48.44 | 108.36 * | 6.04 | 27.30 * | 0.17 | 40.71 * |
| Day 1 – day 15 | 90.51 | 147.49 * | 1.96 | 7.24 * | 16.11 | 32.77 * | -12.04 | -6.76 * | 83.39 | 143.31 * | 17.85 | 39.10 * | 12.01 | 55.71 * |
| Day 2 – day 4 | -34.03 | 20.30 | -2.34 | 2.70 | -12.43 | 3.46 | -4.34 | 0.70 | -24.52 | 32.61 | -9.69 | 10.58 | -10.23 | 29.30 |
| Day 2 – day 3 | -61.76 | -6.10 * | -1.65 | 3.51 | -17.47 | -1.19 * | -2.65 | 2.51 | -56.59 | 1.95 | -18.13 | 2.63 | -15.07 | 24.46 |
| Day 2 – day 5 | -16.71 | 38.96 | -1.05 | 4.11 | -9.76 | 6.52 | -4.05 | 1.11 | -16.89 | 41.65 | -5.59 | 15.17 | -17.99 | 22.07 |
| Day 2 – day 10 | 21.64 | 77.31 * | -0.15 | 5.01 | 0.24 | 16.52 * | -8.15 | -2.99 * | 28.81 | 87.35 * | 8.28 | 29.05 * | 2.72 | 42.26 * |
| Day 2 – day 15 | 66.64 | 122.31 * | 0.75 | 5.91 * | 14.45 | 30.73 * | -12.25 | -7.09 * | 63.76 | 122.30 * | 20.09 | 40.86 * | 14.52 | 57.29 * |
| Day 3 – day 4 | -0.77 | 54.90 | -3.33 | 1.84 | -3.30 | 12.99 | -4.33 | 0.84 | 2.09 | 60.64 * | -2.19 | 18.58 | -15.17 | 24.84 |
| Day 3 – day 5 | 16.56 | 73.54 * | -2.04 | 3.24 | -0.63 | 16.03 | -4.04 | 1.24 | 9.74 | 63.66 * | 1.91 | 23.17 * | -22.93 | 17.61 |
| Day 3 – day 10 | 54.91 | 111.89 * | -1.14 | 4.14 | 9.37 | 26.03 * | -8.14 | -2.86 * | 55.44 | 115.36 * | 15.79 | 37.04 * | -2.21 | 37.80 |
| Day 3 – day 15 | 99.91 | 156.89 * | -0.24 | 5.04 | 23.58 | 40.24 * | -12.24 | -6.96 * | 90.39 | 150.31 * | 27.60 | 48.85 * | 9.60 | 52.82 * |
| Day 4 – day 5 | -9.85 | 45.82 | -1.24 | 3.93 | -5.23 | 11.00 | -2.24 | 2.92 | -20.94 | 37.61 | -6.04 | 14.73 | -27.76 | 12.77 |
| Day 4 – day 10 | 28.50 | 84.17 * | -0.34 | 4.83 | 4.72 | 21.00 * | -6.34 | -1.17 * | 24.77 | 83.31 * | 7.84 | 28.61 * | -7.05 | 32.96 |
| Day 4 – day 15 | 73.50 | 129.17 * | 0.56 | 5.73 * | 18.93 | 35.21 * | -10.44 | -5.27 * | 59.72 | 118.26 * | 19.65 | 40.41 * | 4.77 | 47.98 * |
| Day 5 – day 10 | 9.86 | 66.84 * | -1.74 | 3.54 | 1.67 | 18.33 * | -6.74 | -1.46 * | 15.74 | 75.66 * | 3.25 | 24.50 * | 0.18 | 40.72 * |
| Day 5 – day 15 | 54.86 | 111.84 * | -0.84 | 4.44 | 15.88 | 32.54 * | -10.84 | -5.56 * | 50.69 | 110.61 * | 15.06 | 36.31 * | 12.02 | 55.72 * |
| Day 10 – day 15 | 16.51 | 73.49 * | -1.74 | 3.54 | 5.88 | 22.54 * | -6.74 | -1.46 * | 4.99 | 64.91 * | 1.18 | 22.44 * | -8.19 | 35.03 |

Supplemental Table 6. Lambda for each day of delayed mating as calculated by *popbio* in R.

| Species | Days of Delay in Mating | Lambda |
| --- | --- | --- |
| *T. inclusum* | 1 | 38.41 |
|  | 2 | 5.53 |
|  | 3 | 5.53 |
|  | 4 | 3.45 |
|  | 5 | 3.89 |
|  | 15 | 1.23 |
|  | 29 | 1.07 |
| *T. variabile* | 1 | 44.40 |
|  | 2 | 5.29 |
|  | 3 | 6.45 |
|  | 4 | 3.66 |
|  | 5 | 4.48 |
|  | 10 | 2.73 |
|  | 15 | 1.19 |

SUPPLEMENTAL TABLE 7. Population sizes as projected by the *popbio* package in R for *T. variabile* and *T. inclusum*. Projected 15 generations for each delayed day of mating (1, 2, 3, 4, 5, and 15).

Excel File.
